# Disease activity influences the reclassification of rheumatoid arthritis into very high cardiovascular risk

**DOI:** 10.1186/s13075-021-02542-7

**Published:** 2021-06-04

**Authors:** Iván Ferraz-Amaro, Alfonso Corrales, Juan Carlos Quevedo-Abeledo, Nuria Vegas-Revenga, Ricardo Blanco, Virginia Portilla, Belén Atienza-Mateo, Miguel Á. González-Gay

**Affiliations:** 1 Division of Rheumatology, Hospital Universitario de Canarias, Tenerife, Spain; 2 Epidemiology, Genetics and Atherosclerosis Research Group on Systemic Inflammatory Diseases, Hospital Universitario Marqués de Valdecilla, IDIVAL, Santander, Spain; 3 Division of Rheumatology, Hospital Universitario Marqués de Valdecilla, Universidad de Cantabria, Santander, Spain; 4 Division of Rheumatology, Hospital Doctor Negrín, Las Palmas de Gran Canaria, Spain; 5 Cardiovascular Pathophysiology and Genomics Research Unit, School of Physiology, Faculty of Health Sciences, University of the Witwatersrand, Johannesburg, South Africa

**Keywords:** Rheumatoid arthritis, Cardiovascular disease, Carotid plaque

## Abstract

**Background:**

Previous studies have shown that risk chart algorithms, such as the Systematic Coronary Risk Assessment (SCORE), often underestimate the actual cardiovascular (CV) risk of patients with rheumatoid arthritis (RA). In contrast, carotid ultrasound was found to be useful to identify RA patients at high CV. In the present study, we aimed to determine if specific disease features influence the CV risk reclassification of RA patients assessed by SCORE risk charts and carotid ultrasound.

**Methods:**

1279 RA patients without previous CV events, diabetes, or chronic kidney disease were studied. Disease characteristics including disease activity scores, CV comorbidity, SCORE calculation, and the presence of carotid plaque by carotid ultrasound were assessed. A multivariable regression analysis was performed to evaluate if the reclassification into very high CV risk category was independently associated with specific features of the disease including disease activity. Additionally, a prediction model for reclassification was constructed in RA patients.

**Results:**

After carotid ultrasound assessments, 54% of the patients had carotid plaque and consequently fulfilled definition for very high CV risk. Disease activity was statistically significantly associated with reclassification after fully multivariable analysis. A predictive model containing the presence of dyslipidemia and hypertension, an age exceeding 54 years, and a DAS28-ESR score equal or higher than 2.6 yielded the highest discrimination for reclassification.

**Conclusion:**

Reclassification into very high CV risk after carotid ultrasound assessment occurs in more than the half of patients with RA. This reclassification can be independently explained by the activity of the disease.

## Background

Rheumatoid arthritis (RA) is a chronic systemic inflammatory disease that leads to irreversible joint damage and systemic complications, and the age-adjusted mortality of those affected exceeds that of the general population. This increased risk of premature death in patients with RA is largely due to cardiovascular disease (CVD), particularly coronary artery disease [1]. Increase of carotid artery intima-media wall thickness (cIMT) and higher frequency of carotid plaques have been described in patients with RA [2–5]. Moreover, coronary artery calcification, a finding that is correlated with an increased risk of clinical and angiographic coronary atherosclerosis, is more prevalent in patients with established RA than in patients with early RA or healthy controls [6–8].

Prediction score algorithms for CVD, such as the Systematic Coronary Risk Evaluation (SCORE), was reported to have a suboptimal performance and to underestimate high CV risk in RA [9]. Moreover, according to the 2016 European Guidelines on Cardiovascular Disease Prevention in Clinical Practice [10], carotid artery plaque assessment using ultrasound may be considered a way of reclassifying those patients for whom the SCORE is thought to underestimate their actual CV risk. Although formal reclassification analyses have not been undertaken, it is believed that the presence of carotid plaque may be a modifier of SCORE risk prediction. In keeping with that, a recent 5-year prospective follow-up study has shown that the presence of carotid plaques predicts the development of CV events and death in patients with RA. In this study, the predictable capacity of carotid plaques was higher than that of the SCORE [11].

In the present study, we aimed to determine if specific disease features influence the CV risk reclassification of RA patients assessed by SCORE risk charts and carotid ultrasound.

## Materials and methods

### Study participants

This was a cross-sectional study that included 1279 patients with RA. They were all 18 years or older and were included in the study if they fulfilled the 2010 ACR/EULAR classification criteria for RA [12]. For the purpose of inclusion in the present study, RA disease duration needed to be ≥ 1 year. Patients taking glucocorticoids were included only if they were taking an equivalent dose ≤ 10 mg/day of prednisone. However, patients were excluded if they had diabetes, a history of cancer or any other chronic disease, evidence of active infection, or a glomerular filtration rate < 60 ml/min/1.73 m^2^. The study protocol was approved by the Institutional Review Committees at Hospital Universitario de Canarias, Hospital Doctor Negrín, and Hospital Marqués de Valdecilla, Spain (approval number 17/2012). All subjects provided informed written consent.

### Data collection and laboratory assessments

The subjects completed a CV risk factor and medication use questionnaire and underwent a physical examination. Weight, height, body mass index, abdominal circumference, and systolic and diastolic blood pressure (measured with the participant in a supine position) were assessed under standardized conditions. Information regarding smoking status (current smoker versus non-smoker) and hypertension was obtained from the questionnaire. Medical records were reviewed to ascertain specific diagnoses and medications. Dyslipidemia was defined if one of the following was present: total cholesterol > 200 mg/dl, triglycerides > 150 mg/dl, HDL cholesterol < 40 in men or < 50 mg/dl in women, or LDL cholesterol > 130 mg/dl. Cholesterol, triglycerides, and HDL cholesterol were measured using the enzymatic colorimetric assay. LDL cholesterol was calculated using the Friedewald formula. A standard technique was used to measure the erythrocyte sedimentation rate (ESR) and high-sensitivity C-reactive protein (CRP). Disease activity in patients with RA was measured using the Disease Activity Score (DAS28) in 28 joints [13], the Clinical Disease Activity Index (CDAI) [14], and the Simple Disease Activity Index (SDAI) [15]. Patients with RA were defined as being in clinical remission (DAS28 < 2.6) or having low (DAS28 in the range of 2.6 to 3.2), moderate (DAS28 of > 3.2 to 5.1), or high disease activity (DAS28 > 5.1) as previously described [16]. Function, pain, and patient global estimate of status was measured through the Routine Assessment of Patient Index (RAPID) [17], and disease disability through the Health Assessment Questionnaire (HAQ) score [18].

### Carotid ultrasound assessment

A carotid ultrasound examination was used to assess cIMT in the common carotid artery and to detect focal plaques in the extracranial carotid tree in patients with RA [19]. A commercially available scanner, the Esaote Mylab 70 (Genoa, Italy), equipped with a 7–12-MHz linear transducer and an automated software-guided radiofrequency technique, Quality Intima Media Thickness in real-time (QIMT, Esaote, Maastricht, Holland), was used for this purpose. As previously reported [19], based on the Mannheim consensus, plaque criteria in the accessible extracranial carotid tree (common carotid artery, bulb, and internal carotid artery) were defined as follows: a focal protrusion in the lumen measuring at least cIMT > 1.5 mm, a protrusion at least 50% greater than the surrounding cIMT, or arterial lumen encroaching > 0.5 mm [20].

### Statistical analysis

Demographic and clinical characteristics are shown as frequencies for binary variables.

Continuous variables data are expressed as mean ± standard deviation (SD) or as a median and interquartile range (IQR) for non-normally distributed variables. Patients and controls with carotid plaques based on ultrasound assessment were reclassified into very high-SCORE risk category. Subjects without plaques were maintained in their original SCORE category. cIMT was not used to determine reclassification because according to current guidelines cIMT is not considered an unequivocal CVD on imaging [10]. Univariate differences between reclassified and non-reclassified patients were assessed through Student’s t, Mann-Whitney U, chi-square, or Fisher’s exact tests according to normal distribution or the number of subjects. Logistic regression analysis adjusted for the variables with a *p* value < 0.20 in the univariate analysis was performed to assess the relationship between RA disease-related data and the presence of reclassification. An all-sets logistic regression model was constructed to describe the most parsimonious combination of risk reclassification predictors according to Akaike Information Criteria, Schwarz Bayesian Criterion, area under the curve (AUC), and Hosmer-Lemeshow goodness-of-fit statistics. For characteristics associated with reclassification and that were included in the predictive model, sensitivity versus false-positive frequency (1-specificity) was analyzed using receiver-operating characteristic curves (ROC). To determine the optimal cutoff value of baseline characteristics in predicting reclassification, we calculated the Youden index using the following formula: sensitivity + specificity − 1, with the maximum obtained value corresponding to the optimal cutoff point. To estimate the increase in prediction accuracy between models, we used logistic regression to calculate the ROC curves and the area under the ROC curves (AUC). The SCORE AUC was thus considered the reference and was compared to the other model when adding RA-related data (age, dyslipidemia, hypertension, and DAS28 score). A comparison of ROC curves to test the statistical significance of the difference between the areas under two dependent ROC curves (derived from the same cases) was conducted using the method of DeLong et al. [21]. Reclassification differences between models were studied through the net reclassification index (NRI) and integrated discrimination improvement (IDI) as previously described [22]. Similarly, calibration of the models was calculated using the Hosmer-Lemeshow goodness-of-fit test by grouping individuals on the basis of deciles [23, 24]. All the analyses used a 5% two-sided significance level and were performed using SPSS software, version 24 (IBM Corp.). A *p* value < 0.05 was considered statistically significant.

## Results

### Demographic, laboratory, and disease-related data and SCORE risk category reclassification after carotid sonography

A total of 1279 patients with RA were included in this study. Demographic and disease-related characteristics of the participants are shown in Table 1. Mean ± SD age was 58 ± 13 years and 72% of the patients were female. Current smoking, obesity, dyslipidemia, and hypertension were present in respectively 25, 28, 48, and 37% of the patients. Disease duration was 5 (IQR 2–12) years. RA patients had moderately active disease as shown by the mean DAS28 (3.09 ± 1.49). Half of the patients (50%) were taking prednisone (the median dose of those patients on prednisone was 5 [IQR 4–6] mg/day at the time of the study). Fifty-nine percent of patients were found to be positive for rheumatoid factor and 53% for anti-citrullinated protein antibody (ACPA). Besides, 77% were on disease-modifying antirheumatic drugs and 13% were receiving anti-TNF-alpha therapy. Regarding carotid ultrasound assessment, 53% of the patients with RA had carotid plaques and the average cIMT in patients was 690 ± 140 μm. Additional information regarding RA patient characteristics is shown in Table 1.

**Table 1:** Demographics, cardiovascular risk factors, and disease-related data in RA patients

	RA (*n* = 1279)
Age, years	58 ± 13
Female, n (%)	982 (72)
BMI, kg/m^2^	28 ± 5
Abdominal circumference, cm	94 ± 15
Cardiovascular data	
CV risk factors, n (%)	
Current smoker	326 (25)
Obesity	354 (28)
Dyslipidemia	611 (48)
Hypertension	468 (37)
Diabetes mellitus	0 (0)
Blood pressure, mmHg	
Systolic	131 ± 17
Diastolic	78 ± 10
Lipids	
Total cholesterol, mmol/l	5.28 ± 0.96
Triglycerides, mmol/l	1.27 ± 0.70
HDL cholesterol, mmol/l	1.58 ± 0.44
LDL cholesterol, mmol/l	3.10 ± 0.80
Atherogenic index	3.56 ± 1.06
Statins, n (%)	303 (24)
Aspirin, n (%)	43 (3)
Antihypertensive treatment, n (%)	335 (26)
Disease-related data	
Disease duration, years	5 (2–12)
CRP at time of study, mg/l	2.7 (0.9–6.7)
ESR at time of study, mm/1° h	12 (5–23)
Rheumatoid factor, n (%)	753 (59)
ACPA, n (%)	682 (53)
DAS28-ESR	3.09 ± 1.49
DAS28-PCR	2.99 ± 1.28
SDAI	10 (5–18)
CDAI	9 (4–16)
HAQ	0.750 (0.250–1.250)
RAPID	3.15 ± 2.03
Current drugs, n (%)	
Prednisone	636 (50)
Prednisone doses, mg/day	5 (4–6)
NSAIDs	498 (39)
DMARDs	983 (77)
Methotrexate	730 (57)
Leflunomide	141 (11)
Hydroxychloroquine	282 (22)
Sulphasalazine	44 (3)
Anti-TNF therapy	166 (13)
Adalimumab	56 (4)
Infliximab	18 (1)
Etanercept	66 (5)
Golimumab	5 (0)
Tocilizumab	65 (5)
Rituximab	21 (2)
Abatacept	22 (2)
Baricitinib	12 (1)
Tofacitinib	14 (1)
Historical disease-related data	
History of extraarticular manifestations, n (%)	232 (18)
Erosions, n (%)	453 (35)
CRP at time of disease diagnosis, mg/l	7.0 (2.0–20.0)
CRP > 3 at time of diagnosis, n (%)	685 (54)
ESR at the time of disease diagnosis, mm/1° h	21 (11.38)
Subclinical atherosclerosis	
Carotid IMT, μm	690 ± 140
Carotid plaques, n (%)	678 (53)

Data represent means ± SD or median (IQR) when data were not normally distributed

*CV* cardiovascular, *LDL* low-density lipoprotein, *HDL* high-density lipoprotein, *CRP* C-reactive protein, *NSAID* nonsteroidal anti-inflammatory drugs, *DMARD* disease-modifying antirheumatic drug, *TNF* tumor necrosis factor, *Obesity*, *ESR* erythrocyte sedimentation rate, *BMI* body mass index, *DAS28* Disease Activity Score in 28 joints, *ACPA* anti-citrullinated protein antibodies, *RAPID* routine assessment of patient index, *CDAI* Clinical Disease Activity Index, *SDAI* Simple Disease Activity Index, *HAQ* Health Assessment Questionnaire

Following SCORE risk chart stratification, 533 (42%) patients were included in the low risk category. Contrary, 487 (39%), 139 (11%), and 103 (8%) of the patients were included in the moderate-, high-, and very high-risk categories, respectively. Following carotid ultrasound assessment, 54% of the patients were found to have carotid plaque and consequently were reclassified into the very high SCORE category risk. Specifically, 26% of the patients in the low category, 65% of those in the moderate risk category, and 86% of the ones in the high category moved into the very high SCORE risk category (Table 2).

**Table 2:** SCORE risk category reclassification after carotid ultrasound assessment

		SCORE risk category after carotid ultrasound assessment				% patients reclassified
		Low	Moderate	High	Very high	
Initial SCORE risk category						
Low	533	392			141	26%
Moderate	487		170		317	65%
High	139			19	120	86%
Very high	103				103	-
	1262	392	170	19	681	54%

SCORE risk reclassification data was not available for 17 patients

*SCORE* Systematic Coronary Risk Evaluation

### Differences between reclassified and non-reclassified patients into very high CV risk categories after carotid ultrasound assessment

Several differences were observed in the recorded characteristics of patients with RA who were reclassified following the carotid ultrasound assessment and those who were not reclassified (Table 3). Reclassified patients were older (62 ± 9 vs*.* 54 ± 14 years, *p* = 0.000), and more commonly had hypertension (47% vs*.* 28%, *p* = 0.000) and dyslipidemia (57% vs*.* 40%, *p* = 0.000).

**Table 3:** Multivariable analysis of the differences between reclassified or not reclassified patients

	Not reclassified (*n* = 684)	Reclassified (*n* = 578)	*p*	Reclassification
				OR (95% CI), p*
cIMT, μm	650 ± 140	740 ± 140	**0.000**	
Age, years	54 ± 14	62 ± 9	**0.000**	
Female, n (%)	531 (78)	437 (76)	0.40	
BMI, kg/m^2^	27 ± 6	28 ± 5	0.39	
Abdominal circumference, cm	94 ± 15	94 ± 14	0.92	
Cardiovascular data				
CV risk factors, n (%)				
Current smoker	169 (25)	153 (26)	0.47	
Obesity	193 (28)	155 (27)	0.58	
Dyslipidemia	275 (40)	332 (57)	**0.000**	
Hypertension	192 (28)	271 (47)	**0.000**	
Blood pressure, mmHg				
Systolic	130 ± 18	132 ± 16	**0.024**	
Diastolic	78 ± 10	79 ± 9	0.58	
Lipids				
Total cholesterol, mmol/l	5.26 ± 0.96	5.28 ± 0.96	0.66	
Triglycerides, mmol/l	1.24 ± 0.71	1.28 ± 0.68	0.35	
HDL cholesterol, mmol/l	1.58 ± 0.44	1.58 ± 0.44	0.90	
LDL cholesterol, mmol/l	3.10 ± 0.80	3.10 ± 0.83	0.94	
Atherogenic index	3.56 ± 1.11	3.53 ± 0.97	0.63	
Statins, n (%)	113 (17)	188 (33)	**0.000**	
Aspirin, n (%)	16 (2)	27 (5)	**0.018**	
Antihypertensive treatment, n (%)	140 (20)	190 (33)	**0.000**	
Disease-related data				
Disease duration, years	5 (2–12)	5 (2–12)	0.62	
CRP at time of study, mg/l	2.5 (0.9–6.4)	3.0 (1.0–7.0)	0.71	
ESR at time of study, mm/ 1° hour	11 (5 − 21)	13 (6–25)	**0.007**	1.00 (0.99–1.01), 0.79
Rheumatoid factor, n (%)	380 (56)	360 (62)	**0.019**	**1.58 (1.19–2.11), 0.002**
ACPA, n (%)	352 (51)	320 (55)	0.097	**1.46 (1.09–1.94), 0.011**
DAS28-ESR	2.96 ± 1.50	3.27 ± 1.46	**0.000**	**1.09 (1.02–1.19), 0.028**
DAS28-PCR	2.91 ± 1.28	3.10 ± 1.28	**0.010**	1.04 (0.93–1.16), 0.095
SDAI	3.20 ± 2.05	3.10 ± 2.02	0.73	
CDAI	8 (3–16)	9 (4–17)	0.078	1.00 (0.99–1.02), 0.52
HAQ	0.690 (0250–1.250)	0.750 (0250–1.250)	0.36	
RAPID	3.20 ± 2.05	3.10 ± 2.02	0.61	
Current drugs, n (%)				
Prednisone	330 (48)	291 (50)	0.46	
Prednisone doses, mg/day	5 (2.5–6)	5 (5–7.5)	0.42	
NSAIDs	282 (41)	205 (35)	**0.036**	0.93 (0.70–1.24), 0.62
DMARDs	525 (77)	444 (77)	0.98	
Methotrexate	395 (58)	324 (56)	0.55	
Leflunomide	70 (10)	68 (12)	0.39	
Hydroxychloroquine	147 (21)	132 (23)	0.57	
Sulphasalazine	19 (3)	24 (4)	0.18	1.69 (0.84–3.41), 0.15
Anti-TNF therapy	91 (13)	72 (12)	0.66	
Adalimumab	26 (4)	28 (5)	0.36	
Infliximab	12 (2)	6 (1)	0.29	
Etanercept	40 (6)	26 (4)	0.28	
Golimumab	1 (0)	3 (1)	0.34	
Tocilizumab	37 (5)	27 (5)	0.56	
Rituximab	9 (1)	11 (2)	0.40	
Abatacept	8 (1)	13 (2)	0.14	2.13 (0.77–5.97), 0.15
Baricitinib	5 (1)	6 (1)	0.56	
Tofacitinib	9 (1)	5 (1)	0.45	
Historical disease-related data				
History of extraarticular manif., n (%)	112 (16)	119 (21)	0.058	1.07 (0.75–1.52), 0.72
Erosions, n (%)	230 (34)	216 (37)	**0.048**	1.26 (0.93–1.72), 0.13
CRP at time of disease diagnosis, mg/l	7 (2–19)	8 (3–22)	0.28	
CRP > 3 at time of diagnosis, n (%)	354 (52)	324 (56)	0.068	1.13 (0.78–1.64), 0.52
ESR at the time of diagnosis, mm/1st hour	20 (11–37)	23 (12–40)	0.28	

Data represent means ± SD or median (IQR) when data were not normally distributed

*CV* cardiovascular, *LDL* low-density lipoprotein, *HDL* high-density lipoprotein, *CRP* C-reactive protein, *NSAID* nonsteroidal anti-inflammatory drugs, *DMARD* disease-modifying antirheumatic drug, *TNF* tumor necrosis factor, obesity, *ESR* erythrocyte sedimentation rate, *BMI* body mass index, *DAS28* Disease Activity Score in 28 joints, *ACPA* anti-citrullinated protein antibodies, *RAPID* routine assessment of patient index, *CDAI* Clinical Disease Activity Index, *SDAI* Simple Disease Activity Index, *HAQ* Health Assessment Questionnaire

*Multivariable analysis is adjusted for age, dyslipidemia, hypertension, and statins and aspirin intake

As expected, patients with RA who were reclassified following a carotid ultrasound had greater cIMT than those who were not reclassified (740 ± 140 vs*.* 650 ± 140 μm, *p* = 0.000). However, CRP values did not reveal any differences between reclassified and non-reclassified patients.

Regarding RA-related features, some differences were observed between these two groups of patients (Table 3). Although disease duration was not found to be more frequent in the reclassified patients, both rheumatoid factor and ACPA were more commonly found in the reclassified patients compared to those non-reclassified. The odds ratio (OR) of rheumatoid factor and ACPA for reclassification were, respectively, 1.58 (95% CI 1.19–2.11) and 1.46 (95% CI 1.09–1.94). These associations were observed after multivariable analysis adjusting for age, dyslipidemia, hypertension, and statins and aspirin intake. Additionally, the DAS28-ESR score was statistically significantly associated with reclassification after fully multivariable analysis (OR 1.09 [95% CI 1.02–1.19], *p* = 0.028). It was observed a similar trend for DAS28-CRP and CDAI scores although, in these cases, statistical significance was not reached. Other associations that were significant in the univariable analysis, like those related to the presence of erosions and current use of NSAIDs, did not remain significant after adjustment for covariables (Table 3).

### Predictive model for reclassifying patients into the very high CV risk category following carotid ultrasound assessment

A predictive model was constructed only for those patients with RA who had been included in the low and moderate risk SCORE categories (< 5%) prior to a carotid ultrasound assessment (Table 4). These variables conjointly represented the most parsimonious model capable of predicting the reclassification of patients with RA into the very high CV risk category (Table 4): age, the presence of hypertension and dyslipidemia, and DAS28-ESR score. Moreover, an age exceeding 54 years and a DAS28-ESR score ≥ 2.6 were the cutoffs among the continuous variables that reached the highest Youden indices.

**Table 4:** All the logistic regression model subsets for the prediction of reclassification in patients with RA included in the low and moderate cardiovascular risk category according to the SCORE prior to carotid ultrasound assessment

Variables	OR (95% CI)	*p*	Optimal cutoff	Sensitivity, %	Specificity, %
Age, years	1.11 (1.09–1.13)	**0.000**	54	78	63
Hypertension	1.61 (1.18–2.21)	**0.003**			
Dyslipidemia	1.37 (1.03–1.83)	**0.033**			
DAS28-ESR	1.07 (0.97–1.18)	0.16	2.6	63	46
Pseudo R2	0.188				
AIC	1104				
BIC	1128				
AUC	0.774				
Sensitivity	62%				
Specificity	74%				
pfitHL	0.111				

Values in boldface are statistically significant. *AIC* Akaike information criterion, *BIC* Schwarz Bayesian criterion, *AUC* area under the curve, *pfitHL* Hosmer-Lemeshow goodness-of-fit, *DAS28-ESR* Disease Activity Erythrocyte Sedimentation Rate in 28 joints

Table 5 represents the discrimination, reclassification, and calibration assessment of the model using clinical data (age, dyslipidemia, hypertension, and DAS28-ESR score) versus the reference SCORE model. The SCORE, which included traditional CV risk factors, showed a statistically significant discrimination of reclassification (AUC 0.766, 95% CI 0.737–0.794). The AUC of the model, which contained SCORE plus age, dyslipidemia, hypertension, and DAS28-ESR, was found to have higher discrimination (0.775, 95% CI 0.747–0.803) although statistical significance was not reached (*p* = 0.23). The addition of clinically related data represented a significant change in NRI versus the SCORE reference model (NRI 0.07, 95% CI 0.01–0.12, *p* = 0.018). Similarly, IDI was significantly higher in this model compared to that of the SCORE reference (0.07, 95% CI 0.05–0.08, *p* = 0.000). Model calibration (through a Hosmer-Lemeshow chi-square test) was found to be optimal in the final model (*p* = 0.80).

**Table 5:** Discrimination, reclassification, and calibration assessment of SCORE versus model adding clinical data

	SCORE	SCORE + predictors	*p*
Reclassification in patients with SCORE < 5%			
Discrimination			
AUC	0.766 (0.737–0.794)	0.775 (0.747–0.803)	0.23
Reclassification			
NRI	-	**0.07 (0.01–0.12)**	**0.018**
IDI	-	**0.07 (0.05–0.08)**	**0.000**
Calibration			
H-L test	0.35	0.80	

Clinical data represents age, dyslipidemia, hypertension, and DAS28-ESR

*p* values in AUC rows represent the comparison between both AUC

NRI and IDI are expressed as their values (95% confidence interval) and *p* value

*p* value in H-L test expresses the *p* value of the Hosmer-Lemeshow chi-square statistical test

Predictors are age, hypertension, dyslipidemia, and DAS28-ESR

*SCORE* Systematic Coronary Risk Evaluation, *AUC* area under the curve, *NRI* net reclassification index, *IDI* integrated discrimination improvement

## Discussion

The present work, which included a large series of patients studied by carotid ultrasound, aimed to determine if specific disease features influence the CV risk reclassification of RA patients. It confirmed that reclassification is very common in patients with RA and that disease activity increases the risk for this reclassification. This work also defines several predictors that may be used in the routine clinical practice for the identification of patients with a high probability for being reclassified.

The role of carotid ultrasound in the reclassification of CV risk has been previously studied in other inflammatory diseases. For example, in an earlier work in 343 patients diagnosed with axial spondyloarthritis and 177 controls, patients were more likely reclassified into the very high-risk category than controls after carotid ultrasound [25]. Interestingly, the influence of traditional cardiovascular risk factors on this reclassification was higher in controls compared to patients. However, in this report, although reclassification was associated with higher disease activity and functional and metrological scores, these associations were lost after adjusting for covariables [25]. Similarly, patients with psoriatic arthritis are more frequently reclassified into the very high SCORE risk category following carotid ultrasound assessment than controls [26]. In this case, reclassification was independently explained by the disease activity. Likewise, in a cross-sectional study that included 276 patients with systemic lupus erythematosus, following carotid ultrasound assessment, 32% of the patients were reclassified as very high-risk category [27]. In these SLE patients, disease duration and damage were independently associated with a higher risk of reclassification. The fact that RA shares this higher reclassification frequency with the aforementioned diseases suggests that the SCORE is not a useful instrument for assessing CV risk in patients with RA or other inflammatory diseases. This reclassification can be influenced by the inflammatory activity or damage that these disorders exert.

In our study, a predictive model containing age, two traditional CV risk factors (dyslipidemia and hypertension), and DAS28-ESR higher or equal to 2.6 was capable to show a high discrimination for reclassification. According to our results, in patients with low or moderate CV risk (< 5%), the addition of these predictor variables to SCORE yielded a discrimination higher than that of the SCORE model alone. However, the size effect of this improvement was small and statistical significance was not reached.

In our study, NRI and IDI were significant for the improvement of prediction. For this reason, we believe that the selected variables could be used in the routine clinical practice to choose those patients who, after carotid ultrasound, would probably be reclassified. The fact that our predictive model included not only CV risk factors, but also a variable related to disease activity, supports the fact that reclassification is also driven by factors associated with the disease and not only by factors of traditional CV risk. Interestingly, both rheumatoid factor and ACPA were independently associated with reclassification. This finding is in agreement with previous reports supporting the role of immune dysregulation of autoantibodies in the etiology of CV disease in RA. For example, rheumatoid factor and ACPA have been found to be significant predictors of CV events and mortality in both those with and those without rheumatic diseases [28]. Similarly, in RA, the risk of developing heart failure has been described twice in patients with positive rheumatoid factor than in seronegative patients [29]. ACPA has not been solely linked to RA development and severity, but also plays a role as an additional risk factor in CV disease [30]. It is also believed that citrullination is part of many chronic inflammatory processes and therefore ACPA might act as an independent pro-atherogenic factor in patients with and without RA [31]. Additionally, we understand that in our report probably rheumatoid factor and ACPA are reflecting a subpopulation with higher disease activity which eventually would prone these patients to a higher likelihood of being reclassified.

Interestingly, in our work, both the presence of dyslipidemia and high disease activity were related to the probability of reclassification. It has been recognized that in RA, increased disease activity causes a decrease in lipid-related molecules serum levels. However, it is also known that high levels of LDL cholesterol maintain a positive relation to CV events in patients with RA [32]. That is, the deleterious association between elevated lipid levels and CV events found in the general population is also observed in RA. Therefore, we believe that it is not surprising, and it is completely plausible, to find that both disease activity and the presence of dyslipidemia were related to the probability of reclassification in our study.

We acknowledge the limitation of the cross-sectional nature of the present study that does not allow us to know if patients in whom their risk was reclassified will develop CV events. However, since high/very high CV risk according to the SCORE and the presence of carotid plaque have undoubtedly been linked to CV events, the fact that a patient may be reclassified as having high CV risk is of potential interest. We also acknowledge that some patients were taking CV risk preventive medications such as aspirin, statins, and antihypertensive treatment. We understand that they have probably modified the CV risk of some patients.

## Conclusion

In conclusion, CV risk reclassification after carotid ultrasound is highly frequent in patients with RA. Disease activity is related to this reclassification. We have recently reported that the presence of carotid plaques predicts the development of CV events and death in patients with RA [11]. However, further prospective studies are required to determine the degree of relevance of the additive value of the carotid plaque assessment for the prediction of CVD risk in patients with RA.

## Data Availability

The datasets used and/or analyzed in the present study are available from the corresponding author upon request.

## Abbreviations

ACPA: Anti-citrullinated peptide/protein antibody
AUC: Area under the curve
BMI: Body mass index
CDAI: Clinical Disease Activity Index
CI: Confidence of interval
cIMT: Carotid intima-media thickness
CRP: C-reactive protein
CVD: Cardiovascular disease
DAS28: Disease Activity Score in 28 joints
DMARD: Disease-modifying antirheumatic drug
ESR: Erythrocyte sedimentation rate
HAQ: Health Assessment Questionnaire
HL: Hosmer-Lemeshow
HDL: High-density lipoprotein
IDI: Integrated discrimination improvement
IQR: Inter quartile range
LDL: Low-density lipoprotein
NRI: Net reclassification index
NSAID: Nonsteroidal anti-inflammatory drug
OR: Odds ratio
RAPID: Routine Assessment of Patient Index
RA: Rheumatoid arthritis
ROC: Receiver-operating characteristic curves
SCORE: Systematic Coronary Risk Evaluation
SD: Standard deviation
SDAI: Simplified Disease Activity Index
TNF-α: Tumor necrosis factor-α
